# Supplementary material for: Expression of Recombinant Clostridial Neurotoxin by C. tetani
Source: Microorganisms. 2024 Dec 17;12(12):2611. doi: 10.3390/microorganisms12122611 (PMC11678509; doi:10.3390/microorganisms12122611)
Supplement: Supplementary file 1 [file microorganisms-12-02611-s001.zip › microorganisms-3346470-supplementary.pdf]

Supplemental Materials:

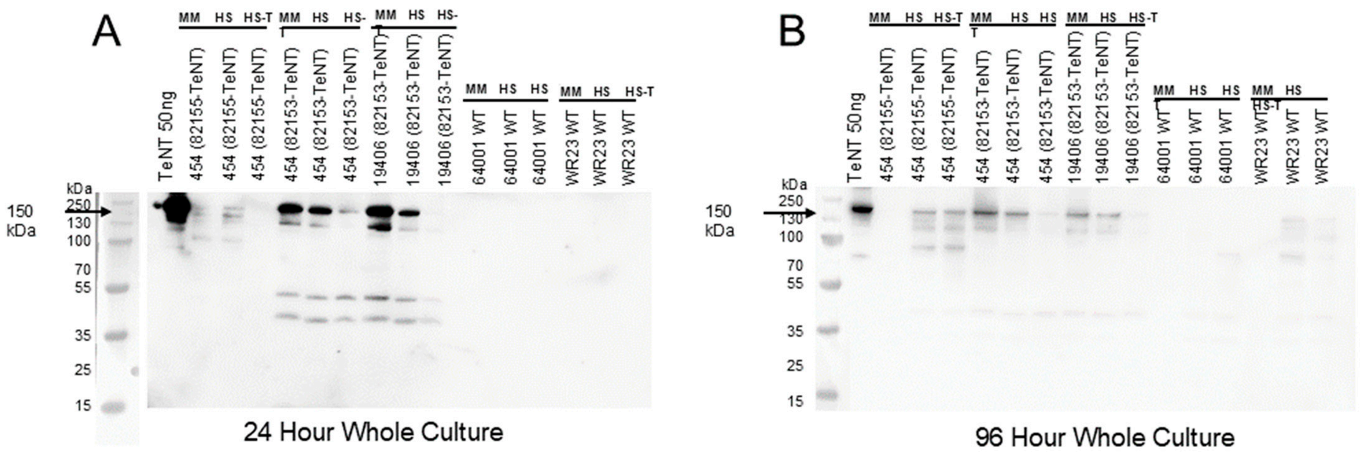

Supplemental Figure S1: Selection of optimal promotor and culture media for expression of recombinant toxins in *C. tetani.* *C. tetani* 454 and *C. tetani* 19406 were conjugated with pMTL82153-TeNT or pMTL82155-TeNT, and cultured statically at 37°C in the noted media alongside wild type *C. tetani* strains 64001 and WR23. 15 ug/mL thiamphenicol was added to *C. tetani* 454 and 19406 growth media for maintenance of the pMTL-TeNT plasmids. 24 hour (A) and 96 hour (B) whole culture, unreduced samples were analyzed by SDS-PAGE and Western blot. 50 ng TeNT 8MTT served as a positive control. MM: modified Mueller Miller (+reduced iron); HS: HySoy (+reduced iron); HS-T: HySoy-T (+reduced iron).

Supplemental Table S1: Antibiotic sensitivity profiles of *C. tetani* and *E. coli* strains

Antibiotic	*C. tetani* 454	*C. tetani* 19406	*E. coli* CA434	*E. coli* S. Express	*E. coli* Interstellar	*E. Coli* TopSex
**Cycloserine (250 µg/mL)**	Resistant	Resistant	Weakly resistant	Weakly resistant	Weakly resistant	Weakly resistant
**Kanamycin (50 µg/mL)**	NT	NT	Resistant	Resistant	Resistant	Resistant
**Spectinomycin (250 µg/mL)**	Weakly resistant	Weakly resistant	NT	Resistant	Resistant	NT
**Thiamphenicol (15 µg/mL)**	Sensitive	Sensitive	Resistant	Resistant	Resistant	Resistant
**Rifampicin (50 µg/mL)**	Sensitive	Sensitive	NT	NT	NT	NT
**Nalidixic Acid (90 µg/mL)**	Sensitive	Sensitive	Sensitive	NT	NT	NT
**Erythromycin (50 µg/mL)**	Sensitive	Sensitive	NT	NT	NT	NT
**Ampicillin (50 µg/mL)**	Sensitive	Sensitive	Sensitive	Sensitive	Sensitive	Sensitive
**Tetracycline (3 µg/mL)**	Sensitive	Sensitive	NT	Resistant	Resistant	NT
**Sulfamethoxazole (30 µg/mL)**	Resistant	Resistant	Resistant	Resistant	Resistant	NT
**Trimethroprim (1.5 µg/mL)**	Resistant	Resistant	Resistant	Resistant	Resistant	NT
**Sulfamethoxazole + Trimethroprim ("co-trimoxazole") (30 µg/mL and 1.5 µg/mL)**	Resistant	Resistant	Weakly resistant	Weakly resistant	Weakly resistant	NT
**Sulfamethoxazole + Trimethroprim + Cycloserine ("TSC") (30 µg/mL, 1.5 µg/mL, 250 µg/mL)**	Resistant	Resistant	Sensitive	Sensitive	Sensitive	NT

NT: Not tested

Weakly resistant: Number of colonies is greatly reduced (<20% compared to control plates)

Supplemental Table S2: Average pH of culture media over time

	pH 24 Hour	pH 48 Hour	pH 96 Hour	pH 144 Hour
C. tetani 454 (82153-TeNT)- Cultured in MM (+ iron)	5.17	5.25	5.38	4.89
C. tetani 454 (82153-TeNT)- Cultured in TPM	4.95	4.78	5.00	4.86
C. tetani 454 (82153-A1ERY)- Cultured in MM (+ iron)	6.71	6.50	6.18	6.15
C. tetani 454 (82153-A1ERY)- Cultured in TPM	6.93	7.17	7.23	7.05
C. tetani 454 (82153) - Cultured in MM (+ iron)	6.80	7.50	7.33	7.18
C. tetani 454 (82153) - Cultured in TPM	6.97	7.12	7.19	7.24
Wild Type *C. tetani* WR23- Cultured in MM (+iron)	6.84	7.50	6.37	6.50
Wild Type *C. tetani* WR23- Cultured in TPM	6.87	7.10	7.18	7.16

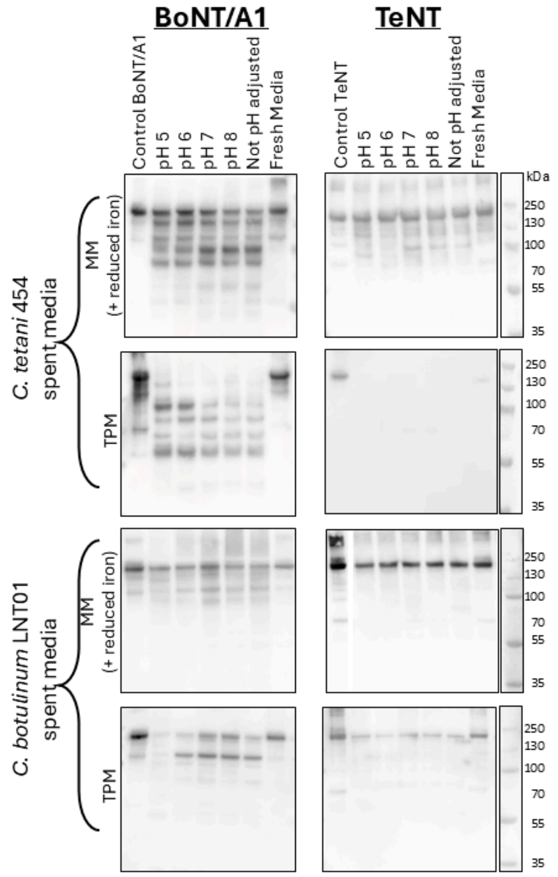

Supplemental Figure S2: Representative Western blots of BoNT/A1 and TeNT stability in spent *C. botulinum* and *C. tetani* media. 3 replicate Western blots per sample condition were analyzed via densitometry to calculate the concentration of ~150 kDa holotoxin remaining after 24 hours of incubation in spent *C. botulinum* LNT01 or *C. tetani* 454 media. The mean percentage of remaining full-length toxin is depicted in Figure 4. These Western blots indicate the extent and size of breakdown products under varying pH conditions.
